# An Ensemble Learning Strategy for Eligibility Criteria Text Classification for Clinical Trial Recruitment: Algorithm Development and Validation

**DOI:** 10.2196/17832

**Published:** 2020-07-01

**Authors:** Kun Zeng, Zhiwei Pan, Yibin Xu, Yingying Qu

**Affiliations:** 1 School of Data and Computer Science Sun Yat-sen University Guangzhou China; 2 School of Computer Science South China Normal University Guangzhou China; 3 School of Business Guangdong University of Foreign Studies Guangzhou China

**Keywords:** Deep learning, Text classification, Ensemble learning, Eligibility criteria, Clinical trial

## Abstract

**Background:**

Eligibility criteria are the main strategy for screening appropriate participants for clinical trials. Automatic analysis of clinical trial eligibility criteria by digital screening, leveraging natural language processing techniques, can improve recruitment efficiency and reduce the costs involved in promoting clinical research.

**Objective:**

We aimed to create a natural language processing model to automatically classify clinical trial eligibility criteria.

**Methods:**

We proposed a classifier for short text eligibility criteria based on ensemble learning, where a set of pretrained models was integrated. The pretrained models included state-of-the-art deep learning methods for training and classification, including Bidirectional Encoder Representations from Transformers (BERT), XLNet, and A Robustly Optimized BERT Pretraining Approach (RoBERTa). The classification results by the integrated models were combined as new features for training a Light Gradient Boosting Machine (LightGBM) model for eligibility criteria classification.

**Results:**

Our proposed method obtained an accuracy of 0.846, a precision of 0.803, and a recall of 0.817 on a standard data set from a shared task of an international conference. The macro F1 value was 0.807, outperforming the state-of-the-art baseline methods on the shared task.

**Conclusions:**

We designed a model for screening short text classification criteria for clinical trials based on multimodel ensemble learning. Through experiments, we concluded that performance was improved significantly with a model ensemble compared to a single model. The introduction of focal loss could reduce the impact of class imbalance to achieve better performance.

## Introduction

Clinical trials are experiments or observations conducted on human volunteers, who are also referred to as subjects in clinical research. Eligibility criteria are the main indicators developed by those conducting the clinical trial to identify whether a subject should be enrolled in a clinical trial [1]. The criteria consist of inclusion and exclusion criteria, which are generally unstructured texts. Recruitment of subjects for clinical trials is generally conducted by manual comparison of their medical records with clinical trial eligibility criteria [2]. In 2009, Thadani et al [3] pointed out that manual comparison was time-consuming, labor-intensive, and inefficient compared with electronic screening. Therefore, clinical trials face many difficulties in recruitment, including difficulty in finding subjects and a long recruitment time [4]. Using natural language processing and machine learning methods to automatically analyze clinical trial eligibility criteria texts and build an automated patient screening system is a promising research topic, with great practical application prospects and clinical value [5,6]. In 2016, Agarwal et al [7] proposed a model to predict the probability of users’ future visits to a medical facility by constructing a matrix of semantic and location-based features from search logs of a search engine.

Text classification is an essential research topic in text information processing. It associates a given text with one or more categories based on characteristics of the text (content, attributes, or features), under a predefined classification taxonomy. Effective feature selection is crucial to the efficiency and accuracy of text classification tasks [8]. Using text classification technology to process medical texts, such as electronic medical records, not only improves the work efficiency of medical institutions [3], but also provides a basis for the further processing of medical text data. In addition, text classification technology has great significance for the research of knowledge graph construction [9], question answering system design [10], and automatic text summary [11].

However, unlike open domains, the complexity of medical texts makes it extremely difficult to classify them. First, the complexity of medical texts mainly comes from a large number of domain-specific terms. Different categories of texts correspond to medical terms of disease names, drug names, body part names, and other information, which presents difficulties in text segmentation and subsequent text feature extraction [12]. Second, the diversity of medical natural language texts also increases the complexity of medical text classification [13]. For example, a disease concept may have more than 10 mentions in a disease category. In addition, this type of medical text data is generally imbalanced, which presents difficulties in the classification of categories that contain a small amount of data [14].

With the rapid development of deep learning [15], many short text classification methods based on word vector models have emerged. Kaljahi et al [16] proposed the Any-gram kernel method to extract N-gram features of short texts, and used a bidirectional long-term and short-term memory network (BILSTM) to classify the texts. The method made improvements in topic- and sentence-level sentiment analysis tasks. Kim et al [17] used convolutional neural networks (CNN) to solve sentence classification problems. Lee et al [18] combined recurrent neural networks (RNN) and convolutional neural networks to classify short texts. Hsu et al [19] mixed convolutional neural networks with recurrent neural networks and proposed a structure-independent gate representation algorithm for sentence classification. Zhou et al [20] introduced a 2-dimensional maximum pooling operation to a bidirectional long-term and short-term memory network (BILSTM) to extract the features of texts in the temporal and spatial dimensions in a text classification task. In recent years, the Bidirectional Encoder Representations from Transformers (BERT) model [21] proposed by Google utilized a self-attention mechanism transformer [22], which improved feature extraction capability based on a long-term and short-term memory network (LSTM) and improved the bidirectional fusion function in stitching mode.

In order to solve the difficulties (eg, feature extraction) caused by a large number of domain specific diseases, medicines, body parts names, and other terminology, our paper proposed a character-level short text classification model. For word embedding, 4 character-level word embedding models were selected: BERT, A Robustly Optimized BERT Pretraining Approach (RoBERTa), XLNet, and Enhanced Representation through Knowledge Integration (ERNIE). We used a pretrained model based on Chinese corpus to accelerate the convergence of the model. In order to reduce the data imbalance problem, focal loss was introduced to the training process to train the model more stably. Finally, LightGBM was used to ensemble the 4 models to improve overall performance.

The main contributions of this paper are as follows: (1) a character-level ensemble learning model created by integrating BERT, RoBERTa, XLNet, and ERNIE was proposed for eligibility criteria text classification. (2) The focal loss as a loss function was leveraged to solve the problem of data imbalance among different categories. (3) The evaluation results showed that our ensemble learning model outperformed several baseline methods, demonstrating its effectiveness in the eligibility criteria text classification task.

## Methods

### Data Set

Our data set comes from the evaluation task of the China Health Information Processing Conference (CHIP) 2019. There are three evaluation tasks. The first task is the standardization of clinical terms [23]. The main goal of this task is to standardize the semantics of surgical entity mentions in Chinese electronic medical records. Given a surgical word, the corresponding standardized word is required. The second task is disease question transfer learning [24]. The main objective is to perform transfer learning between diseases based on Chinese disease question and answer data. Specifically, given question pairs in five different disease types, it is required to determine whether the semantics of two questions are the same or similar. The third evaluation task is the short text classification of clinical trial eligibility criteria.

The data set contains 38341 clinical trial eligibility criteria texts and has been manually annotated by human experts. Table 1 shows some specific examples of eligibility criteria texts and their annotated categories. For instance, the corresponding category of “血糖<2.7 mmol/L” (blood glucose<2.7 mmol/L) is “Laboratory Examinations.”

The data set contains 44 various categories of clinical trial eligibility criteria in total, including “Disease,” “Multiple,” “Therapy or Surgery,” etc. The data set is further divided into a training set, a validation set, and a test set. The training set contains 22962 pieces of eligibility criteria texts, while the validation and test sets contain 7682 and 7697 texts, respectively.

**Table 1:** Examples of eligibility criteria texts and corresponding annotated categories.

Eligibility criteria text	Annotated category
年龄>80岁 (Age>80)	Age
近期颅内或椎管内手术史 (Recent intracranial or spinal canal surgery)	Therapy or surgery
血糖<2.7 mmol/L (Blood glucose<2.7 mmol/L)	Laboratory examinations
2)性别不限,年龄18～70岁 (Unlimited gender, aged 18-70 years)	Multiple
合并造血系统或恶性肿瘤等严重原发性疾病 (A serious primary disease, such as one involving the hematopoietic system or a malignant tumor)	Disease
其他研究者认为不适合参加本研究的患者 (Patients that are unsuitable for this study that were considered by other investigators)	Researcher decision
预期生存超过12周 (Expected survival over 12 weeks)	Life expectancy
男、女不限 (Male or female)	Gender

### Overall Framework

**Figure 1:**
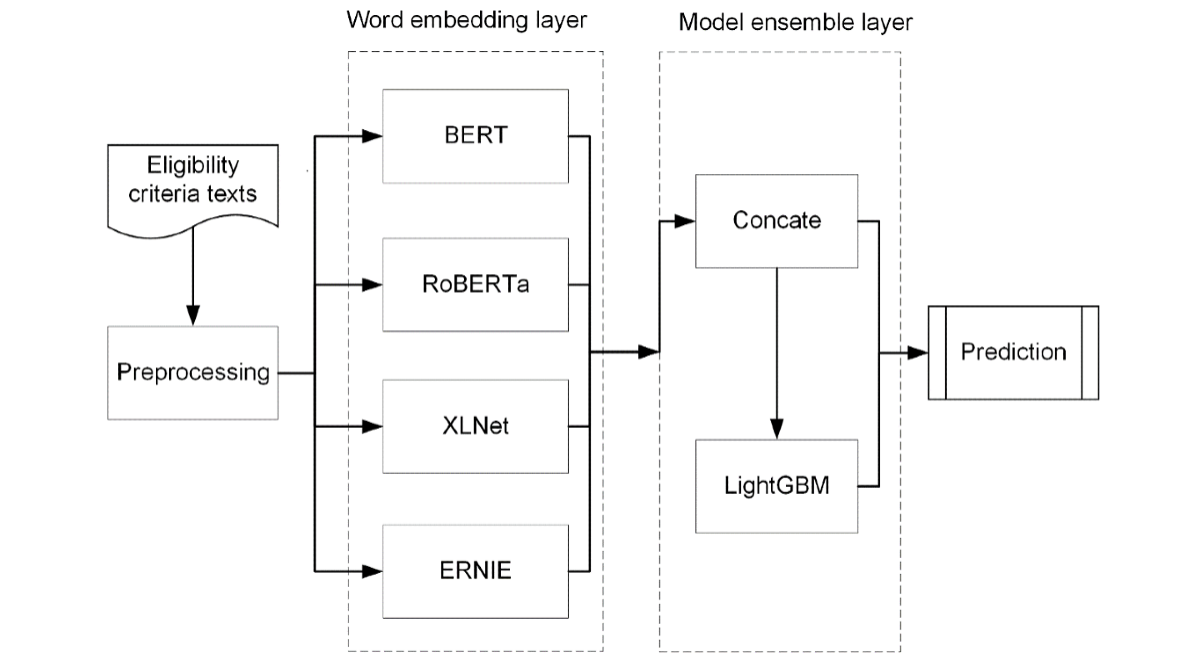
The framework of the proposed model that contains two layers: a word embedding layer consisting of 4 pretrained models (BERT, XLNet, ERNIE, and RoBERTa); and a model ensemble layer containing LightGBM, used to learn information by combining the outputs of the 4 pretrained models. BERT: Bidirectional Encoder Representations from Transformers; ERNIE: Enhanced Representation through Knowledge Integration; LightGBM: Light Gradient Boosting Machine; RoBERTa: A Robustly Optimized BERT Pretraining Approach.

The overall framework of our proposed model is shown in Figure 1. As shown in the flowchart, the sample texts were preprocessed and converted from characters to numeric vectors for training. After that, we used BERT, RoBERTa, XLNet, and ERNIE to train the vectors, and calculated the Softmax value for the results of each model. Finally, we used LightGBM for model ensemble training.

Most existing text representation methods are based on words, phrases, sentences, or analysis of semantic and grammatical structure in texts. However, existing word segmentation techniques are not suitable in the medical field due to complex grammatical structures. Therefore, we use character-level textual representations to avoid these problems. Accordingly, our model is based on the mainstream character-level text models described below.

### BERT and RoBERTa

BERT [21] stands for Bidirectional Encoder Representation from Transformers. BERT introduces Masked Language Modeling (LM), which masks and predicts tokens in the corpus, and uses transformers [22] as an encoder to extract the contextual features of texts. The features are promoted to sentence level through sentence-level negative sampling [25], learning sentence and sentence pair representation.

Moreover, RoBERTa [26] uses dynamic masking on the basis of BERT. It removes the Next Sentence Prediction (NSP) mechanism in the pretraining process, and uses larger data for training to make RoBERTa more robust.

In this paper, we use a pretrained model based on Chinese BERT and RoBERTa with a Whole Word Masking (WWM) version [27]. In our preprocessing, a “[CLS]” symbol is added before input texts. It uses the transformer to extract features from texts and encode global information. The output of highest hidden layer at the “[CLS]” position is taken as a sentence-level feature. Subsequently, a fully connected layer is used to output text classification probability values.

### Preprocessing

In natural language processing tasks, data preprocessing often greatly impacts the final result. The purpose of data preprocessing is to improve the quality of extracted text features [28]. In addition to the preprocessing carried out for different models mentioned above, we also applied some text preprocessing. First, we used regular expressions on input sentences to reduce noise characters in sentences. Subsequently, a stop word list is utilized to remove meaningless words. For sentences longer than 40 characters, we use the first 40 characters for training. To normalize input vectors, we used a dictionary to map each character to a corresponding value, and convert texts into a vector composed of numerical values.

### ERNIE

Based on BERT, ERNIE [29] pretrains the model by masking semantic units such as words and entity concepts in masked LM, and enhances the semantic representation capabilities by introducing multisource data corpora.

We used a Chinese corpus–based pretrained model named ERNIE. In our preprocessing, a “[CLS]” symbol is added before input texts, and the features are extracted through a transformer with unshared weight. Here, global information is encoded into “[CLS].” Finally, we take the output of the highest hidden layer at “[CLS]” as a sentence-level feature for text classification by a fully connected layer.

### XLNet

XLNet is an autoregressive language model created by Google Brain and Carnegie Mellon University, which avoids the shortcomings of the BERT model in training-tuning differences caused by using masks not existing in real texts and ignoring the relevance of cover words in prediction.

We used the XLNet [30] pretraining model based on a Chinese corpus. Text features were extracted by using Transformer-XL. The output of pooling of the highest hidden layer is used as the sentence-level feature for text classification.

### Model Ensemble

In the last layer of our model, after obtaining the training output of BERT, ERNIE, XLNet, and RoBERTa, we performed Softmax processing to obtain the probability that each submodel predicts 44 labels for each text. Let the probability of each model output be 
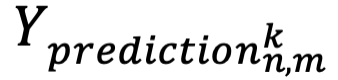
 where k ∈ [1,4] represents 4 submodels, n represents the size of the training set text, and m represents that each text prediction corresponds to m different categories, set to 44 in our model. Matrix row splicing is conducted on these 4 probability variables and they are merged into a matrix of n rows and 4m columns of 
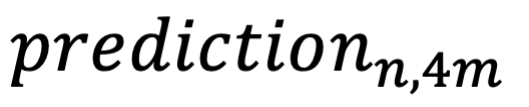
 as a training set. Using the idea of linear weighting, we take the training set and actual labels of n texts as input and use the LightGBM [31] to train our model to achieve the final predictions of each text.

### Class Imbalance and Loss Function

**Figure 2:**
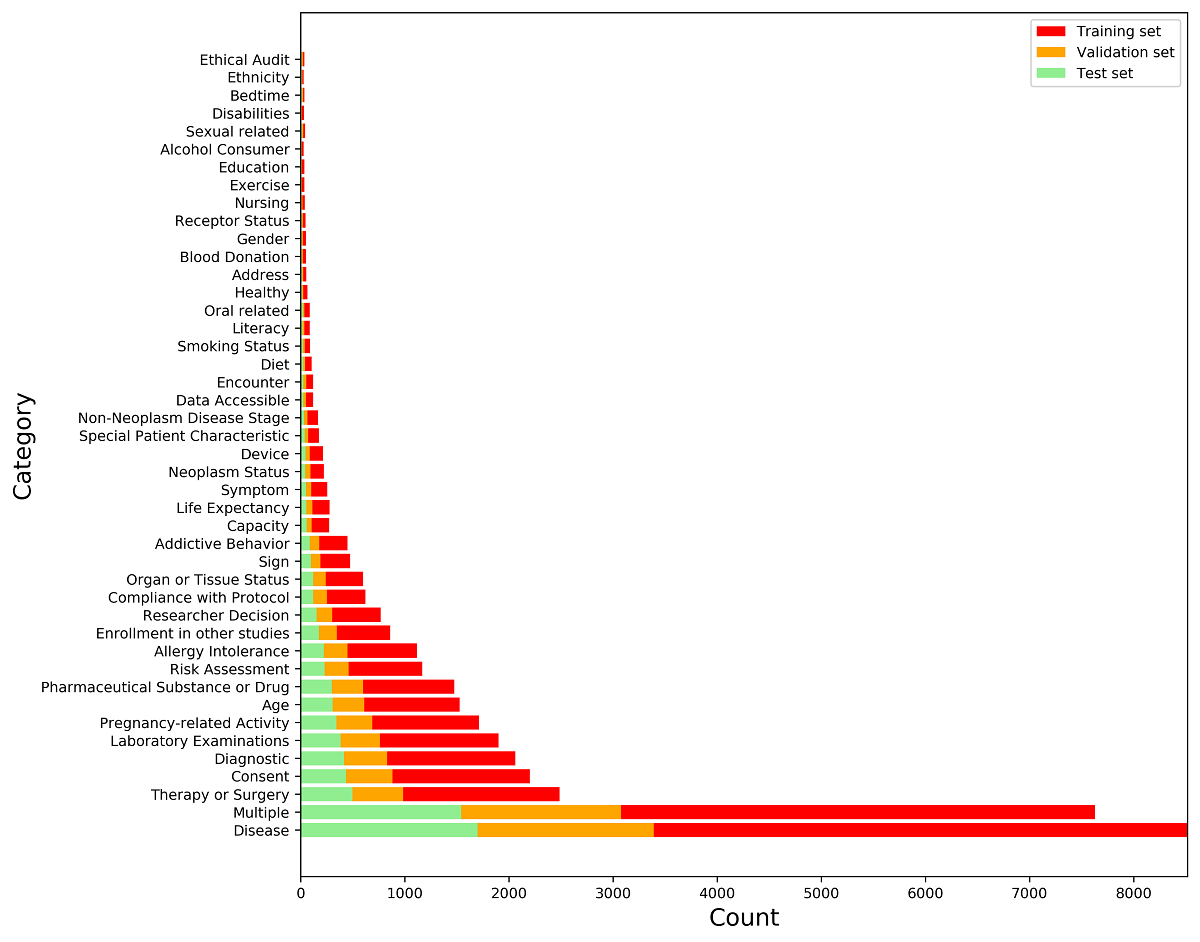
Histogram distributions of the training set, validation set, and test set. The y-axis represents different labels, and the x-axis represents quantity.

We calculated the statistical characteristics of the training, validation, and test sets, and identified that there is a data imbalance issue. Figure 2 summarizes the distributions of categories on the training, test, and validation sets to illustrate the distribution of numbers in each class. As indicated by the distribution of each data set, the data in each category is significantly unbalanced. The largest category is “Disease,” with a total of 8518 samples, and the smallest category is “Ethnicity,” with only 29 samples.

To solve the problem of data imbalance, we applied focal loss [32] as the loss function for training. We compared the classification of focal loss with the popular cross-entropy loss (CE loss) in the next section to show the advantage of focal loss. Supposing the expression of *p_t_* is the following:


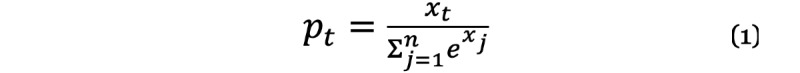


*x_t_* is the score on category *t*, and *p_t_* is the prediction probability of an input sample on category *t*. The expression of CE loss is calculated using Equation 2.


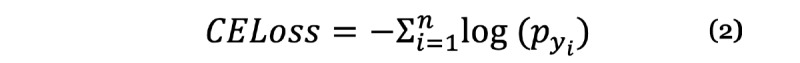



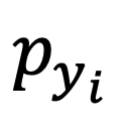
 represents the category of the *i*-th input sample. After that, we introduced the expression of focal loss as shown in Equation 3, where γ is a parameter. The value of γ was empirically set to 2 in this paper.


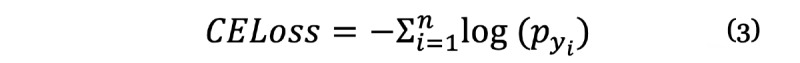


### Experiment Setup

In order to ensure the reproducibility of test results and to facilitate the experimental comparison of different methods, this experiment fixed the random number seed to 0, the batch size was 128, and model parameters remained the same as the learning rate was set to 2×10^-5^.

Our training used an NVIDIA 2080Ti graphics card. The memory size was 11 GB. Due to limited video memory, BERT, XLNet, and ERNIE were trained separately, including the training set (22932 pieces of data), the validation set (7652 pieces of data), and test set (7697 pieces of data). The learning rate was 2×10^-5^, and 30 rounds of training were conducted for each model using Adam as an optimizer.

Our model was implemented using Python, based on the open source framework of PyTorch and open source pretraining parameters. To make the model converge faster and obtain better performance, we used open source parameters trained with a large amount of Chinese texts for different models for transfer learning.

### Evaluation Metrics

To evaluate our model, we applied four commonly used metrics in machine learning. They are accuracy, precision, recall, and F1 score. These four metrics are also often used in classification tasks in deep learning. F1 is the standard metric for this task; it combines precision and recall. Macro F1 is a parameter index that can best reflect the effectiveness and stability of the model. According to the task requirement of CHIP 2019, we applied the macro average on these four metrics. The calculation of the four metrics is as shown in Equations 4-7:


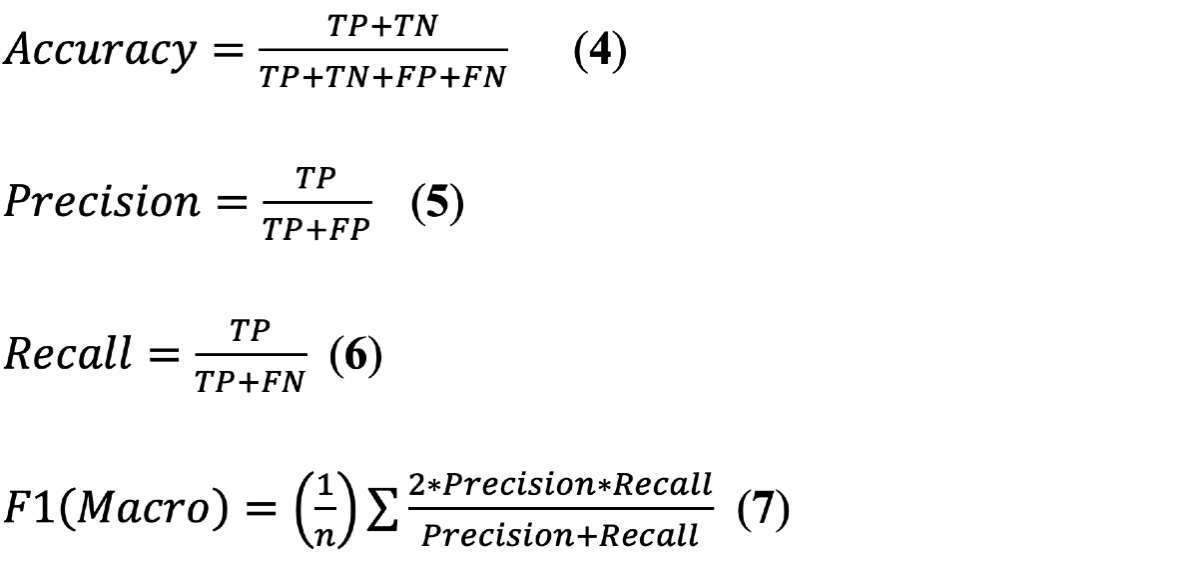


TP (true positive) is the number of categories *t* that were correctly predicted as *t*. FP (false positive) is the number of categories that were not *t* and were wrongly predicted as *t*. FN (false negative) is the number of categories that were *t* and the model wrongly predicted it as another class. TN (true negative) is the number of categories that were not *t* and were correctly predicted as another class. In Equations 4-7, n denotes the number of categories, which is 44 in this paper.

## Results

We used the current 4 single models for experiments and each model was tested on the training set only, to provide baselines for comparison. Table 2 presents the results of the 4 single models and the 2 fusion methods of Voting and LightGBM. The results from using the multimodel fusion methods were higher than that of the single models by an average of 2.35%.

**Figure 3:**
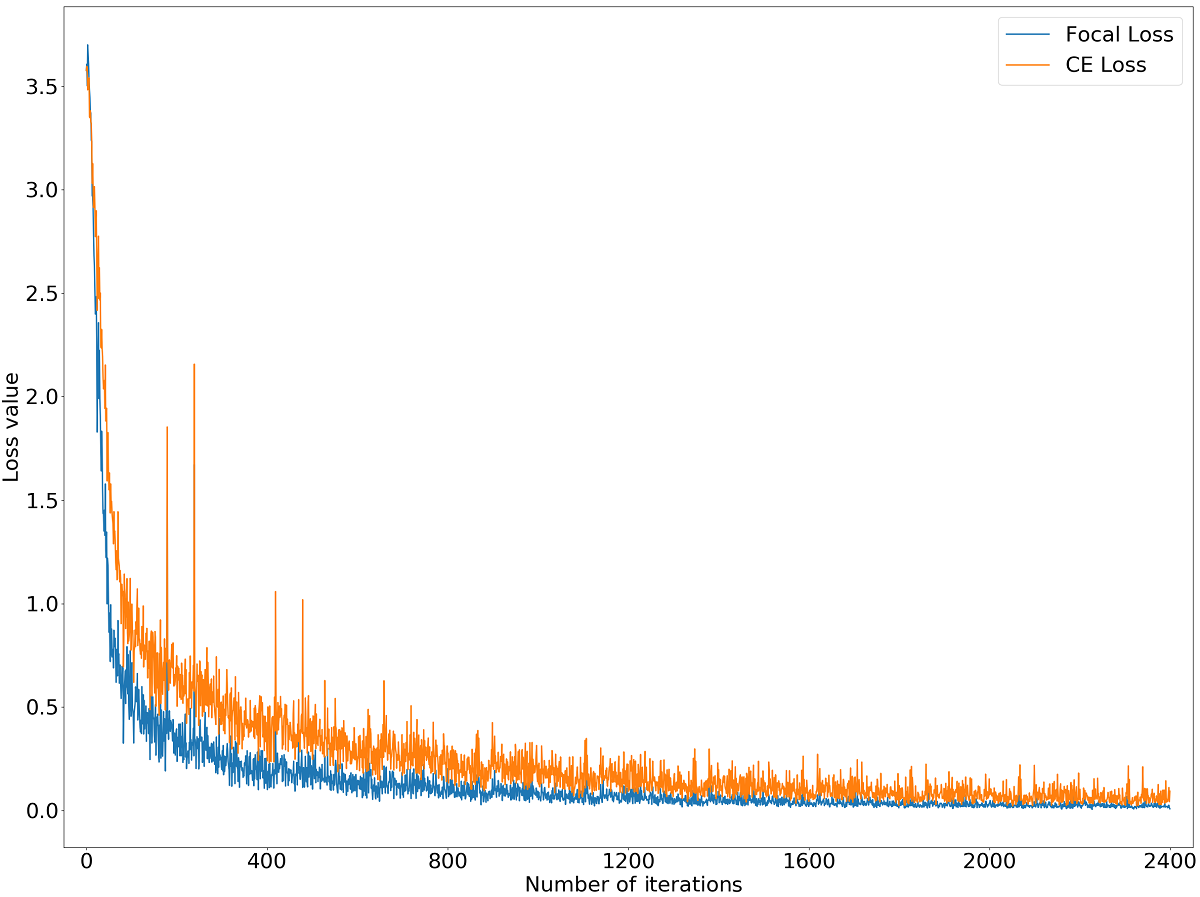
Histogram distributions of the training set, validation set, and test set. The y-axis represents different labels, and the x-axis represents quantity.

By studying the loss function of the training set, we found that the performance of a single model using focal loss was significantly better using than CE loss for data sets with unbalanced categories. Figure 3 shows the convergence of loss function on the training set, in which the convergence of focal loss on the training set was faster and the value of loss function fluctuated slightly. Thus, the training speed was more stable.

Due to the structure and parameter differences of the models, the probability distributions of the models were different from each other. For a classification task, the final parameter distributions of the models were varied, and the results from different inputs had different confidences. After model assembling, a more accurate prediction result of the input sample [33] was acquired. To determine whether the performance of the classification models was limited by the amount of data, we kept the training set unchanged and randomly reduced the data volume of each category in the training set (not verification set) by 25% to keep the same data distribution. Experiments on the stability of the models were performed separately. The results are shown in Table 3.

**Table 2:** The performance of our model and baseline models using the full training data set.

Model	Accuracy	Precision	Recall	Macro F1
BERT^a^	0.836	0.779	0.802	0.788
XLNet	0.844	0.790	0.811	0.795
ERNIE^b^	0.836	0.786	0.795	0.783
RoBERTa^c^	0.840	0.791	0.800	0.792
Ensemble (Voting)	0.846	0.800	0.812	0.802
Our model	0.846	0.803	0.817	0.808

^a^BERT: Bidirectional Encoder Representations from Transformers.

^b^ERNIE: Enhanced Representation through Knowledge Integration.

^c^RoBERTa: A Robustly Optimized BERT Pretraining Approach.

**Table 3:** The performance of the 6 models using the reduced training data set.

Model	Accuracy	Precision	Recall	Macro F1
BERT^a^	0.831	0.781	0.776	0.771
XLNet	0.839	0.797	0.759	0.773
ERNIE^b^	0.822	0.754	0.765	0.751
RoBERTa^c^	0.832	0.7952	0.770	0.776
Ensemble (Voting)	0.832	0.795	0.770	0.776
Our model	0.834	0.790	0.785	0.780

^a^BERT: Bidirectional Encoder Representations from Transformers.

^b^ERNIE: Enhanced Representation through Knowledge Integration.

^c^RoBERTa: A Robustly Optimized BERT Pretraining Approach.

## Discussion

### Limitations

There was a limitation of the proposed method. Compared with the performance of the model under the complete data volume (Table 2), the performance of each model after reducing unequal data volume (Table 3) was significantly lower than that of the entire data volume. The F1 score of the BERT model decreased by 2.16%; the F1 score of the XLNet model decreased by 2.77%; and the F1 score of the model we proposed decreased by 3.47%. Therefore, insufficient training data is an important factor limiting model performance.

### Future Work

In the future, we believe that two aspects of our model could be improved: the data and the model. Short text has the characteristic of having fewer words, and may not be able to provide enough information [34]. Therefore, a pretrained model in the medical field that was pretrained by medical corpus will benefit the stability of the model [35]. In addition, effective data enhancement could be applied on short text data to enhance text features and improve results.

### Conclusions

The classification of clinical trial eligibility criteria texts is a fundamental and critical step in clinical target population recruitment. This research proposed an ensemble learning method that integrates the current cutting-edge deep learning models BERT, ERNIE, XLNet, and RoBERTa. Through model ensemble in two layers, we trained our model and compared it with a list of baseline deep learning models on a publicly available standard data set. The results demonstrated that our proposed ensemble learning method outperformed the baseline methods by 2.35% on average.

## Abbreviations

BERT: Bidirectional Encoder Representations from Transformers.
BILSTM: Bidirectional Long Short-Term Memory.
CHIP: China Health Information Processing Conference.
CNN: convolutional neural network.
ERNIE: Enhanced Representation through Knowledge Integration.
LightGBM: Light Gradient Boosting Machine.
LSTM: Long Short-Term Memory.
NLP: Natural Language Processing.
NSP: Next Sentence Prediction.
RNN: recurrent neural network.
RoBERTa: A Robustly Optimized BERT Pretraining Approach.
WWM: Whole Word Masking.
